# Ganglioside GM1 Contributes to the State of Insulin Resistance in Senescent Human Arterial Endothelial Cells*

**DOI:** 10.1074/jbc.M115.684274

**Published:** 2015-09-02

**Authors:** Norihiko Sasaki, Yoko Itakura, Masashi Toyoda

**Affiliations:** From the Research Team for Geriatric Medicine (Vascular Medicine), Tokyo Metropolitan Institute of Gerontology, Sakaecho 35-2, Itabashi-ku, Tokyo 173-0015, Japan

**Keywords:** aging, endothelial cell, endothelial dysfunction, ganglioside, insulin resistance, senescence, GM1

## Abstract

Vascular endothelial cells (ECs) play central roles in physiologically important functions of blood vessels and contribute to the maintenance of vascular integrity. Therefore, it is considered that the impairment of EC functions leads to the development of vascular diseases. However, the molecular mechanisms of the EC dysfunctions that accompany senescence and aging have not yet been clarified. The carbohydrate antigens carried by glycoconjugates (*e.g*. glycoproteins, glycosphingolipids, and proteoglycans) mainly present on the cell surface serve not only as marker molecules but also as functional molecules. In this study, we have investigated the abundance and functional roles of glycosphingolipids in human ECs during senescence and aging. Among glycosphingolipids, ganglioside GM1 was highly expressed in abundance on the surface of replicatively and prematurely senescent ECs and also of ECs derived from an elderly subject. Insulin signaling, which regulates important functions of ECs, is impaired in senescent and aged ECs. Actually, by down-regulating GM1 on senescent ECs and overloading exogenous GM1 onto non-senescent ECs, we showed that an increased abundance of GM1 functionally contributes to the impairment of insulin signaling in ECs. Taken together, these findings provide the first evidence that GM1 increases in abundance on the cell surface of ECs under the conditions of cellular senescence and aging and causes insulin resistance in ECs. GM1 may be an attractive target for the detection, prevention, and therapy of insulin resistance and related vascular diseases, particularly in older people.

## Teaser

**Background:** The roles of gangliosides in senescent endothelial cells (ECs) were unknown.

**Results:** Ganglioside GM1 increased on senescent and aged ECs. GM1 contributes to the impairment of insulin signaling in ECs.

**Conclusion:** Increased GM1 with senescence and aging contributes to insulin resistance in ECs.

**Significance:** GM1 is the first known contributor to insulin resistance in ECs.

## Introduction

Vascular endothelial cells (ECs)^2^ constitute the endothelium of blood vessels, which forms an interface between the blood and the vessel wall and plays important roles in vascular homeostatic functions. Excessive activation or dysfunction of ECs is considered to lead to the development of vascular-related diseases, including restenosis, arteriosclerosis, and cancer (1). Insulin signaling regulates important functions in ECs and contributes to the maintenance of vascular integrity. For example, insulin signaling in ECs modulates NO production by endothelial NO synthase (eNOS) activation and the expression of adhesion molecules, and it also attenuates the progression of atherosclerosis (2, 3). So, insulin resistance in ECs, which is a dysfunction characterized by the impairment of insulin signaling, is considered to lead to the initiation and progression of vascular and vascular-related diseases (4, 5).

The aging of the population worldwide is resulting in increasing numbers of older people, among whom vascular disease is the leading cause of death. Senescence and aging of ECs have been considered to increase the risk of vascular diseases (6, 7). Therefore, it is important to clarify the mechanisms underlying senescence and aging-associated diseases to lower the risk for vascular disease and extend healthy life expectancy. From previous research, it is known that senescence and aging cause EC dysfunctions such as reduced NO production and elevated inflammation (8). Consequently, it has been speculated that senescence and aging produce insulin resistance in ECs, but until now, the molecular mechanisms of the insulin resistance that occurs with senescence and aging have been unclear. To investigate these mechanisms, we focused on glycosphingolipids (GSLs).

GSLs are composed of a glycan structure attached to a lipid tail containing the sphingolipid ceramide. GSLs are widely expressed on cell membranes in lower and higher eukaryotic organisms. GSLs have frequently been used as important developmental marker molecules and have been suggested to have important biological functions (9, 10). So far, studies on gangliosides (molecules composed of GSLs with one or more sialic acids) related to senescence and aging have been reported in neural tissues but not yet in ECs (11, 12). It has been demonstrated that gangliosides are fine regulators of receptor tyrosine kinases signaling, including insulin signaling, and that changing cell surface ganglioside compositions in physiopathological conditions results in altered cellular responses (13, 14). In 3T3-L1 adipocytes, the monosialodihexosylganglioside (GM3) was found to contribute to insulin resistance in pathological conditions such as obesity (15). Furthermore, it was shown that potent inhibitors of GSLs improve ganglioside-mediated insulin sensitivity in pathological model mice (16, 17). Therefore, it is considered that gangliosides play important roles in insulin resistance. However, the remaining issues are as follows: (i) whether gangliosides contribute to insulin resistance in ECs and (ii) what kinds of gangliosides contribute to the dysfunction.

We focused on gangliosides of ECs, and we hypothesized that changes in the abundance of cell surface gangliosides with senescence and aging contribute to EC dysfunctions such as insulin resistance. In this study, we revealed for the first time that monosialotetrahexosylganglioside (GM1) among the several gangliosides species was increased in abundance on the cell surface of ECs with cellular senescence (replicative and premature) and aging and that the increased abundance of GM1 resulted in a state of insulin resistance in senescent ECs.

## Experimental Procedures

### 

#### 

##### Cell Culture

Human aortic endothelial cells (HAECs) were purchased from a commercial vendor (Lonza, Walkersville, MD). These cells were characterized by positive staining for acetylated LDL and von Willebrand factor and negative staining for smooth muscle actin, according to the manufacturer's product sheet. In the initial culture (baseline), these cells did not express the smooth muscle marker, smooth muscle myosin heavy chain. Cells were obtained from a 27-year-old male (HAECs-young), a 40-year-old male (HAECs-middle), and an 81-year-old male (HAECs-elder). Experiments were mainly performed using HAECs-middle, and some experiments were performed using the other HAECs. Cells were grown in endothelial growth medium-2 (EGM-2) that was supplemented with growth factors, antibiotics, and 2% fetal bovine serum (EGM-2 SingleQuot; Lonza). Cells were passaged at 80% confluence and seeded at a density of 2,500–5,000 cells/cm^2^. All cells were studied at a confluence of 75–80%. Population doubling levels (PDLs) were calculated at each passage using the following equation: *n* = (log2*X* − log2*Y*) (where *n* = the PDL; *X* = the number of cells at the end of one passage; and *Y* = the number of cells that were seeded at the beginning of one passage). The PDL at the first plating of a newly purchased cell stock was defined as PDL 0. In this study, cells (HAECs-middle) with PDL 11–13 (average, 11 ± 1) and PDL 19–20 (average: 19 ± 1) were used as early-passage (non-senescent) control cells and late-passage (senescent-induced) cells, respectively. For the induction of premature senescence, early-passage HAECs at about 75–80% confluence were exposed for 2 h to 250 μm hydrogen peroxide (H_2_O_2_) diluted in HAEC culture medium. The cells were washed three times with PBS to remove H_2_O_2_ and re-cultured in fresh culture medium for 72 h to allow senescent characteristics to be exhibited. For the reduction of ganglioside levels, HAECs were treated with either vehicle (ethanol) only or 10 μm *N*-(5′-adamantane-1′-yl-methoxy)-pentyl-1-deoxynojirimycin (AMP-dNM) (Cayman Chemical, Ann Arbor, MI) throughout the culture period until they were used for experiments. For the incubation of cells with exogenous GM1, early-passage HAECs were cultured with either vehicle (methanol) only or 50 nm GM1 (Sigma) in HAEC culture medium for 24 h.

##### Senescence-associated β-Galactosidase (SA-β-Gal) Assay

SA-β-Gal activity was assayed by using a senescence detection kit (BioVision Inc., Milpitas, CA) according to the manufacturer's instructions. Briefly, cells were washed twice with PBS, exposed to fixation solution for 10 min, and then incubated overnight in freshly prepared staining solution. After staining, cells were counterstained with DAPI, and then microscopic examination of about four fields of view was performed. By counting the number of SA-β-Gal-positive cells based on their blue color and the total number of cells stained with DAPI using ImageJ software (National Institutes of Health, Bethesda), the percentage of SA-β-Gal-positive cells was calculated to estimate the percentage of senescent cells.

##### FACS Analysis

In general, trypsinization reduces the abundance of some cell surface antigens. Therefore, to avoid this effect, cells were harvested with Accutase® cell detachment solution (Merck Millipore, Billerica, MA), and these dissociated single cells were incubated with primary antibodies diluted in FACS buffer (0.5% (w/w) BSA and 0.1% (w/w) sodium azide in PBS) for 30 min on ice. After washing, the cell suspension was incubated with Alexa Fluor® 488- or Alexa Fluor® 647-conjugated secondary antibodies (Molecular Probes, Eugene, OR) diluted in FACS buffer for 30 min on ice. For the detection of GM1, cells were incubated with Alexa Fluor® 647-conjugated cholera toxin B subunit (Molecular Probes) diluted in FACS buffer (0.5% BSA and 0.1% sodium azide in PBS) for 30 min on ice. Cell sorting and analysis were performed using a FACSAria^TM^ Cell Sorter (BD Biosciences). We used the following primary antibodies: anti-GM3 (NBT Laboratories Inc., Tokyo, Japan); anti-monosialotrihexosylceramide (GM2) (TCI, Tokyo, Japan); anti-monosialotrihexosylceramide (GD1a) (TCI); anti-ganglioside GD3 (Merck Millipore); anti-ganglioside GD2 (TCI); anti-ganglioside GD1b (TCI); and anti-insulin receptor (IR)α (Abcam, Cambridge, UK).

##### Analysis of Proteins by Immunoblotting

For the observation of insulin signaling, the cell culture medium was replaced with EGM-2 containing 1% (v/v) FBS for 6 h, and the cells were stimulated for 5 min with 1 μm human insulin (Wako, Osaka, Japan). Cells were lysed with lysis buffer (50 mm Tris-HCl, pH 7.4, 150 mm NaCl, and 1% (v/v) Triton^TM^ X-100) containing protease and phosphatase inhibitor mixtures (Roche Applied Science). For immunoprecipitation, cells were lysed with lysis buffer (50 mm Tris-HCl, pH 7.4, 150 mm NaCl, 1.5 mm MgCl_2_, 5 mm EDTA, and 1% Triton^TM^ X-100) containing protease and phosphatase inhibitor mixtures, and immunoprecipitations were performed with the appropriate antibody and protein G magnetic beads (Veritas, Tokyo, Japan). Control immunoprecipitations were performed using the same antibodies heat-inactivated for 10 min at 95 °C before use. Samples prepared as described above were separated by SDS-PAGE using a gel of the appropriate percentage and then transferred onto PVDF membranes (Merck Millipore). After blocking, the membranes were incubated with the following primary antibodies: monoclonal rabbit anti-Akt (4691; Cell Signaling Technology, Danvers, MA); monoclonal rabbit anti-phosphorylated Akt (Ser-473; 4060; Cell Signaling Technology); monoclonal mouse anti-eNOS (610297; BD Biosciences); monoclonal rabbit anti-phosphorylated eNOS (Ser-1177; 9570; Cell Signaling Technology); polyclonal rabbit anti-IRα (ab5500; Abcam); polyclonal rabbit anti-IRβ (ab131238; Abcam); polyclonal rabbit anti-IR substrate (IRS)1 (2382; Cell Signaling Technology); monoclonal rabbit anti-IRS2 (ab52606; Abcam); and monoclonal mouse anti-β-actin (A5316; Sigma). The membranes were then incubated with the appropriate peroxidase-conjugated secondary antibodies (Cell Signaling Technology), washed, and developed with ECL^TM^ prime reagents (GE Healthcare). For the detection of GM1, dot blot analysis was performed using HRP-conjugated cholera toxin B subunit (Molecular Probes).

##### Immunostaining

Cells were fixed with 4% (w/v) paraformaldehyde and washed, and then the non-permeabilized cells were double-stained with an anti-IRα antibody and Alexa Fluor® 594-conjugated cholera toxin B subunit (Molecular Probes). After washing, cells were permeabilized with chilled methanol for 10 min at 4 °C and subsequently blocked with PBS containing 1% (w/v) BSA and 5% (v/v) normal goat serum. After washing, cells were incubated with primary antibodies, anti-p16^INK4a^ (Abcam), or isotype control at 4 °C overnight. After washing, cells were stained with an Alexa Fluor® 488-conjugated secondary antibody (Molecular Probes) and an Alexa Fluor® 647-conjugated secondary antibody (Molecular Probes) and then counterstained with DAPI. Immunofluorescence images were taken using a fluorescence microscope (Leica Microsystems, Wetzlar, Germany).

##### Real Time PCR

Total RNA was isolated from cells using a High Pure RNA isolation kit (Roche Applied Science) and subsequently reverse-transcribed using a ReverTra Ace® qPCR RT kit (Toyobo, Osaka, Japan). Real time PCR was performed using a Power SYBR® Green kit (Applied Biosystems, Foster City, CA) using a StepOnePlus^TM^ real time PCR system (Applied Biosystems). Primer sets for real time PCR are listed in Table 1.

**TABLE 1:** **List of primer sets for real time PCR**

Gene	Forward primer	Reverse primer
*ST3GAL5*	AGGAATGTCGTCCCAAGTTTG	GGAGTAAGTCCACGCTATACCT
*B4GALNT1*	ACAGCAGACACAGTCCGGTTCT	GCGGGTGTCTTATGCGGATA
*ST8SIA1*	TACTCTCTCTTCCCACAGG	GACAAAGGAGGGAGATTGC
*B3GALT4*	GAAGGAGGCCAGGTTTTGC	CCCGGCCCAAGTACAGAAG
*ST3GAL2*	TGGACGGGCACAACTTCA	TGCCAACATCCTGCTCAAAG
*NEU3*	AATGTGAAGTGGCAGAGGTGA	TCACAGAGCTGTCGACTCAGG
*IR*	CGAAGATTTCCGAGACCTCAGT	TCGAAGATGACCAGCGCGTAG
*IRS1*	GCAACCAGAGTGCCAAAGTGA	GGAGAAAGTCTCGGAGCTATGC
*IRS2*	GCAGAACATCCACGAGACCAT	GGAACTCGAAGAGCTCCTTGAG
β-Actin	GGTCATCACCATTGGCAATGAG	TACAGGTCTTTGCGGATGTCC

##### Statistics

Western blot images were densitometrically analyzed by ImageJ software (National Institutes of Health). Values were expressed as means ± S.D. from basically three independent experiments. Independent samples of Student's *t* tests were used for statistical data analysis with Excel.

## Results

### 

#### 

##### Ganglioside GM1 Is Increased in Abundance on Senescent ECs

**FIGURE 1.**
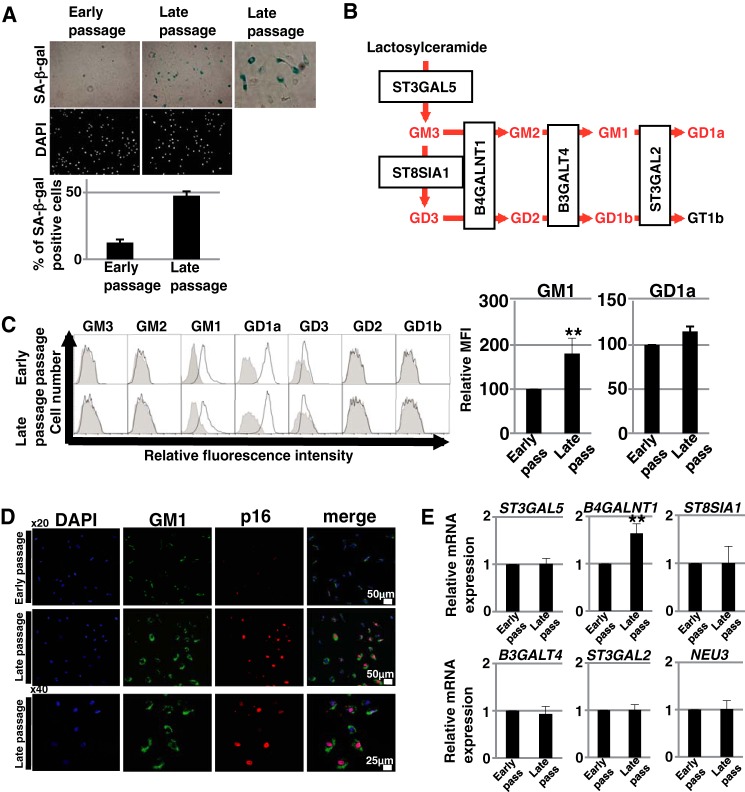
**Increase of GM1 on replicatively senescent ECs.** *A,* early- and late-passage HAECs were stained for SA-β-Gal activity, and SA-β-Gal-positive cells were quantitated as a percentage of total cells. Results are presented as means ± S.D. from three independent experiments. Representative images of staining for SA-β-Gal and DAPI are shown. *B*, main synthetic pathway of gangliosides is shown. Gangliosides shown in *red font* were examined in this study. Glycosyltransferases contributing to each synthetic pathway are also shown. *C*, cell surface abundances of gangliosides in early- and late-passage HAECs were analyzed by flow cytometry using antibodies against each ganglioside. Three independent experiments were performed, and representative results are shown. Controls are presented by *thinner lines* with *gray color*. MFIs relative to those of early-passage HAECs are shown on the *right-hand side*. Results are presented as means ± S.D. from six (GM1) or three independent experiments. **, *p* < 0.01. *D*, immunocytochemical staining was performed in early- and late-passage HAECs. Representative images are shown (p16, *red*; GM1, *green*; DAPI, *blue*). *E*, real time PCR analysis of the glycosyltransferases shown in *B* and *NEU3* was performed using cDNA derived from early- and late-passage HAECs. The results are shown after normalization against the values obtained for early-passage HAECs (value = 1). Results are presented as means ± S.D. from five independent experiments. **, *p* < 0.01.

It is well known that the amount and composition of gangliosides in the cell membrane can change depending on the cellular condition, such as the developmental and pathological state (9). Changes in membrane gangliosides have been shown particularly in neural tissues during the induction of senescence (11). To identify cell surface gangliosides involved in senescence of ECs, we performed FACS analysis of early- and late-passage HAECs. Late-passage HAECs exhibited an enlarged and flattened morphology, a lowered proliferative capacity (0.07 ± 0.03 *versus* 0.36 ± 0.04 PDLs/day), and an increased amount of SA-β-gal-positive cells compared with that of early-passage HAECs (Fig. 1*A*). These results indicated that replicative senescence was induced in late-passage HAECs. FACS analysis of gangliosides (Fig. 1*B*) revealed that GM1 and GD1a were mainly present on HAECs (Fig. 1*C*). Furthermore, as shown in Fig. 1*C*, the abundance of GM1 was significantly increased on late-passage HAECs compared with that of early-passage HAECs. In contrast, GD1a did not significantly change in abundance with increasing passage number (Fig. 1*C*). Additionally, the majority of senescent cells, which were characterized by large cell size (measured by forward scatter) and/or high autofluorescence (measured by emission intensity in the fluorescein isothiocyanate channel) (18), exhibited a high abundance of GM1 (data not shown). These populations were confirmed as senescent by SA-β-Gal assay (data not shown). Immunocytochemical staining showed that the abundance of GM1 as well as the expression of the senescence marker p16^INK4a^ were increased in late-passage HAECs (Fig. 1*D*). This result confirmed that the abundance of GM1 was increased in replicatively senescent HAECs. To elucidate the mechanisms contributing to the increased abundance of GM1 during replicative senescence, we analyzed the expression levels of glycosyltransferases and sialidase (*NEU3*) involved in the ganglioside synthetic pathways. Real time PCR analysis showed that the expression of *B4GALNT1*, which catalyzes the synthesis of GM2, was increased in late-passage HAECs compared with that of early-passage HAECs (Fig. 1*E*).

**FIGURE 2.**
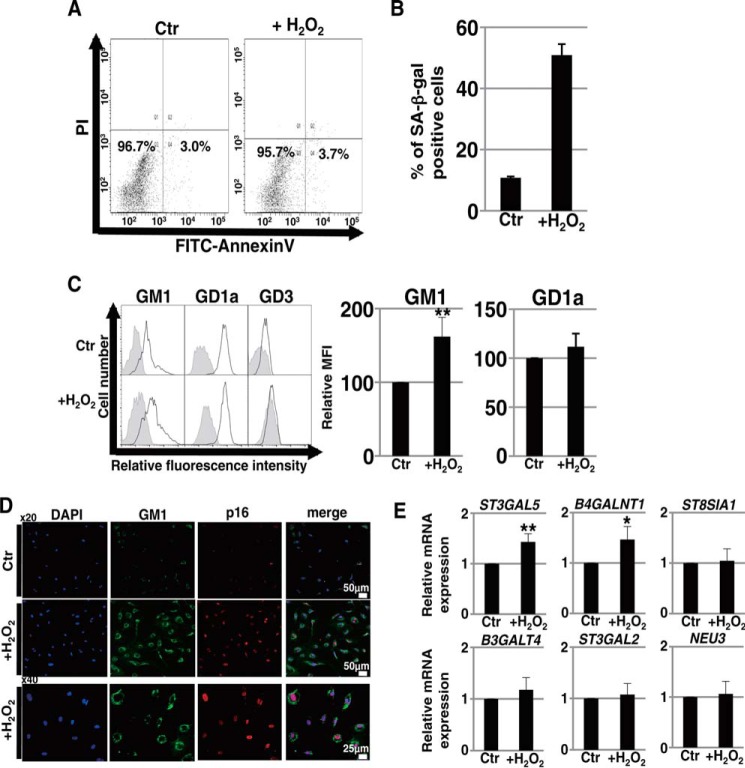
**Increase of GM1 on H_2_O_2_-induced prematurely senescent ECs.** *A*, measurement of apoptotic cells was performed in ECs 72 h after treatment with 250 μm H_2_O_2_ by using an annexin V-biotin apoptosis detection kit (BioVision Inc.) according to the manufacturer's instructions. A representative histogram obtained by flow cytometry analysis is shown. *B*, non-senescent and H_2_O_2_-induced prematurely senescent HAECs were stained for SA-β-Gal activity, and SA-β-Gal-positive cells were quantitated as a percentage of total cells. Results are presented as means ± S.D. from three independent experiments. *C*, cell surface abundances of gangliosides in non-senescent and H_2_O_2_-induced prematurely senescent HAECs were analyzed by flow cytometry using antibodies against each ganglioside, and representative results are shown. Controls are presented by *thinner lines* with *gray color*. MFIs relative to those of non-senescent HAECs are shown. Results are presented as means ± S.D. from six (GM1) or three independent experiments. **, *p* < 0.01. *D*, immunocytochemical staining was performed in non-senescent and H_2_O_2_-induced prematurely senescent HAECs. Representative images are shown (p16, *red*; GM1, *green*; DAPI, *blue*). *E*, real time PCR analysis of the glycosyltransferases shown in Fig. 1*B* and *NEU3* was performed using cDNA derived from non-senescent and H_2_O_2_-induced prematurely senescent HAECs. The results are shown after normalization against the values obtained for non-senescent HAECs (value = 1). Results are presented as means and S.D. of five independent experiments. Significant differences are indicated: *, *p* < 0.05; **, *p* < 0.01. Control (*Ctr*): untreated cells (non-senescent).

Another type of senescence termed “premature senescence” can be induced in the absence of detectable telomere loss by a variety of conditions (19). H_2_O_2_, a reactive oxygen species implicated in vascular disease and cancer, is a known inducer of premature senescence through the oxidative stress pathway when delivered at a subcytotoxic dose (20). Conversely, a high dose of H_2_O_2_ is known to induce EC apoptosis (21). For this reason, we first determined appropriate concentrations of H_2_O_2_ for the induction of premature senescence in HAECs. Exposure to concentrations of >350 μm H_2_O_2_ induced apoptosis (data not shown), but exposure to 250 μm H_2_O_2_ did not (Fig. 2*A*). Treatment with 250 μm H_2_O_2_ induced premature senescence as determined by the SA-β-Gal assay (Fig. 2*B*). Then, we investigated whether gangliosides were involved in the premature senescence of HAECs. In prematurely senescent HAECs, FACS analysis showed that the abundance of GM1 was significantly increased (Fig. 2*C*) and also that the majority of the senescent populations characterized by large cell size and/or high autofluorescence exhibited a high abundance of GM1 (data not shown). Furthermore, immunocytochemical staining confirmed that the abundance of GM1 was increased in prematurely senescent HAECs, accompanied by increased p16^INK4a^ expression (Fig. 2*D*). Among the glycosyltransferases and sialidase involved in the GM1 synthetic pathways, real time PCR analysis showed that the expression levels of *B4GALNT1* and also *ST3GAL5*, which catalyze the synthesis of GM3, were increased in prematurely senescent HAECs (Fig. 2*E*). Taken together, these results demonstrate that GM1 was increased in abundance on the cell surface of HAECs with both replicative and premature senescence.

##### Insulin Signaling Is Impaired in Senescent ECs

**FIGURE 3.**
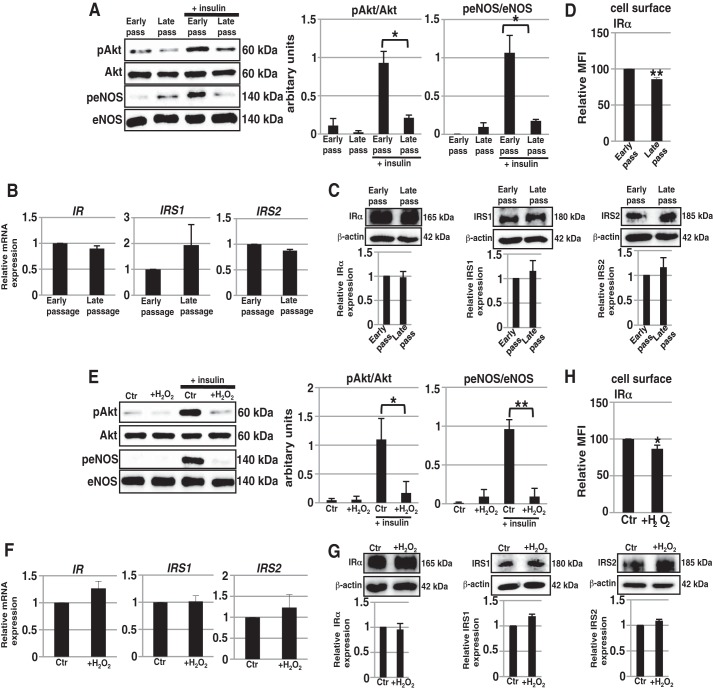
**Down-regulation of insulin signaling in senescent ECs.** Western blot analysis for insulin signaling was performed in early- and late-passage HAECs (*A*) and in non-senescent and H_2_O_2_-induced prematurely senescent HAECs (*E*). The histograms show mean densitometric readings ± S.D. for the phosphorylated proteins normalized to those of the loading controls. Real time PCR analysis of *IR*, *IRS1*, and *IRS2* was performed using cDNA derived from early- and late-passage HAECs (*B*) and non-senescent and H_2_O_2_-induced prematurely senescent HAECs (*F*). The results shown were normalized against the values obtained for early-passage, non-senescent HAECs (value = 1). Western blot analysis for IRα, IRS1, and IRS2 was performed in early- and late-passage HAECs (*C*) and in non-senescent and H_2_O_2_-induced prematurely senescent HAECs (*G*). The histograms show mean densitometric readings ± S.D. of IRα, IRS1, IRS2, and loading controls (β-actin). FACS analysis for cell surface IRα was performed in early- and late-passage HAECs (*D*) and in non-senescent and H_2_O_2_-induced prematurely senescent HAECs (*H*). MFIs relative to that of early-passage or non-senescent HAECs are shown. All values were obtained from three independent experiments; *, *p* < 0.05; **, *p* < 0.01. Control (*Ctr*): untreated cells (non-senescent).

Insulin signaling is important for EC functions such as NO production and the regulation of the expression of adhesion molecules, which are protective against the development of vascular diseases. Therefore, it is considered that insulin resistance in ECs is a major risk factor for vascular diseases. It has been demonstrated that a state of insulin resistance is produced in ECs under clinical conditions such as obesity and hyperglycemia (5, 22). However, whether insulin resistance occurs as a dysfunction in senescent ECs has not yet been clarified. We first examined insulin signaling in replicatively senescent HAECs. The insulin signaling cascade activates protein kinase B (Akt), which in turn phosphorylates and activates eNOS in ECs (23). Western blot analysis showed that the insulin-induced phosphorylation of Akt and eNOS was not increased in late-passage HAECs compared with that in early-passage HAECs, indicating that insulin signaling was impaired in replicatively senescent HAECs (Fig. 3*A*). To elucidate the mechanism of insulin resistance in senescent HAECs, we investigated the expression levels of insulin signaling molecules, such as the IR and IRS. There were no significant differences in the mRNA or protein expression levels of IR and IRS between early- and late-passage HAECs (Fig. 3, *B* and *C*). Only cell surface levels of IR were slightly reduced in late-passage HAECs (Fig. 3*D*). Next, we examined insulin signaling in prematurely senescent HAECs in a similar way. As shown in Fig. 3*E*, prematurely senescent HAECs also exhibited insulin resistance. Furthermore, the expression levels of IR and IRS were also not significantly changed after the induction of premature senescence, except for slight reduction in cell surface IR expression (Fig. 3, *F–H*). Thus, it has been demonstrated that insulin resistance developed in both replicatively and prematurely senescent ECs.

##### GSL Synthesis Inhibitor Restores the Defect of Insulin Signaling in Senescent ECs

**FIGURE 4.**
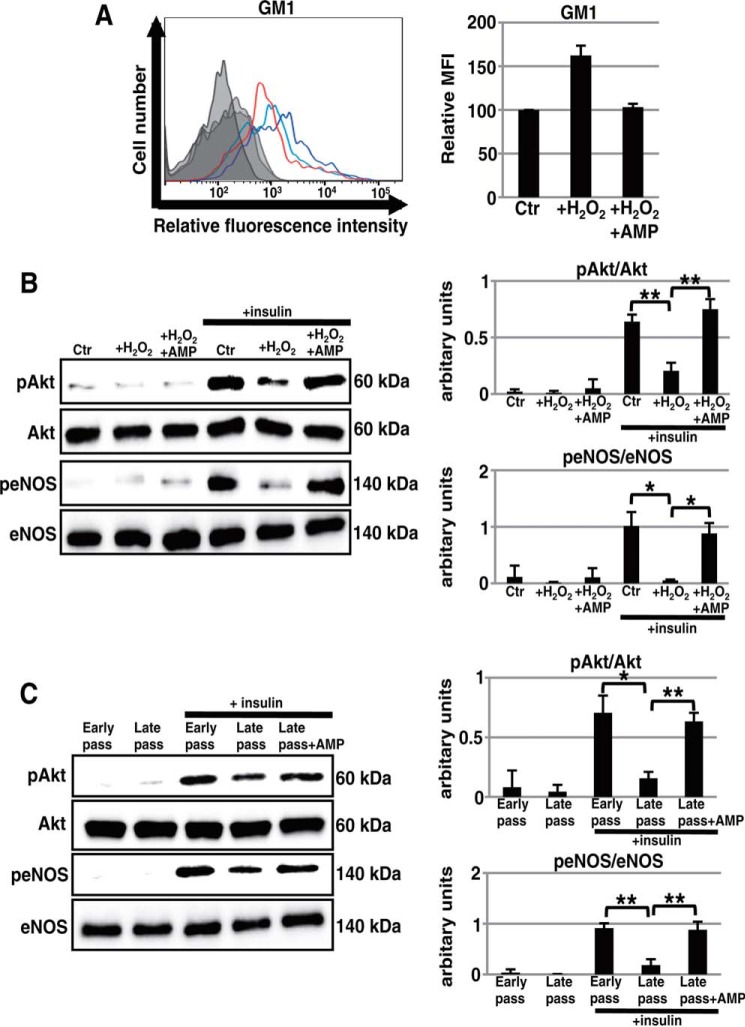
**Restoration of defective insulin signaling in senescent ECs after treatment with a glycosphingolipid synthesis inhibitor.** *A*, cell surface abundance of GM1 in non-senescent and H_2_O_2_-induced prematurely senescent HAECs with or without AMP-dNM treatment for 3 days was analyzed by flow cytometry. Three independent experiments were performed, and a representative overlaid histogram is shown on the *left-hand side* (*red, dark blue*, and *light blue lines* represent results for non-senescent HAECs, H_2_O_2_-induced prematurely senescent HAECs without AMP-dNM treatment, and those with treatment, respectively). Controls are presented by *thinner lines* with *gray* color. MFIs relative to that of non-senescent HAECs of three independent experiments are shown. Western blot analysis for insulin signaling was performed in non-senescent and H_2_O_2_-induced prematurely senescent HAECs (*B*) and in early- and late-passage HAECs (*C*) with and without AMP-dNM treatment for 3 days. The *histograms* show mean densitometric readings ± S.D. of the phosphorylated proteins normalized to those of the loading controls. Values were obtained from three independent experiments; *, *p* < 0.05; **, *p* < 0.01. Control (*Ctr*): untreated cells (non-senescent).

So far it has been shown that increased abundances of GM1 and GM3 in the cell membrane contribute to insulin resistance (15, 24). To elucidate the underlying mechanism of insulin resistance in senescent ECs, we examined the possible contribution of increased GM1 to insulin resistance. For this purpose, we used AMP-dNM. AMP-dNM is a specific inhibitor of glucosylceramide synthase that can be used to study the functional roles of endogenous gangliosides without affecting ceramide levels (16, 25). Treatment with AMP-dNM lowered the increased abundance of GM1 on prematurely senescent HAECs to the same level as that detected in non-senescent HAECs (Fig. 4*A*). Western blot analysis showed that the impairment of insulin signaling in prematurely senescent HAECs was restored by AMP-dNM treatment (Fig. 4*B*). These results indicate that an increased abundance of GM1 on prematurely senescent HAECs contributes to insulin resistance. In addition, restoration of insulin signaling was observed in AMP-dNM-treated late-passage HAECs (Fig. 4*C*). Thus, these results indicate that an increased abundance of GM1 contributes to insulin resistance in both replicatively and prematurely senescent ECs.

**FIGURE 5.**
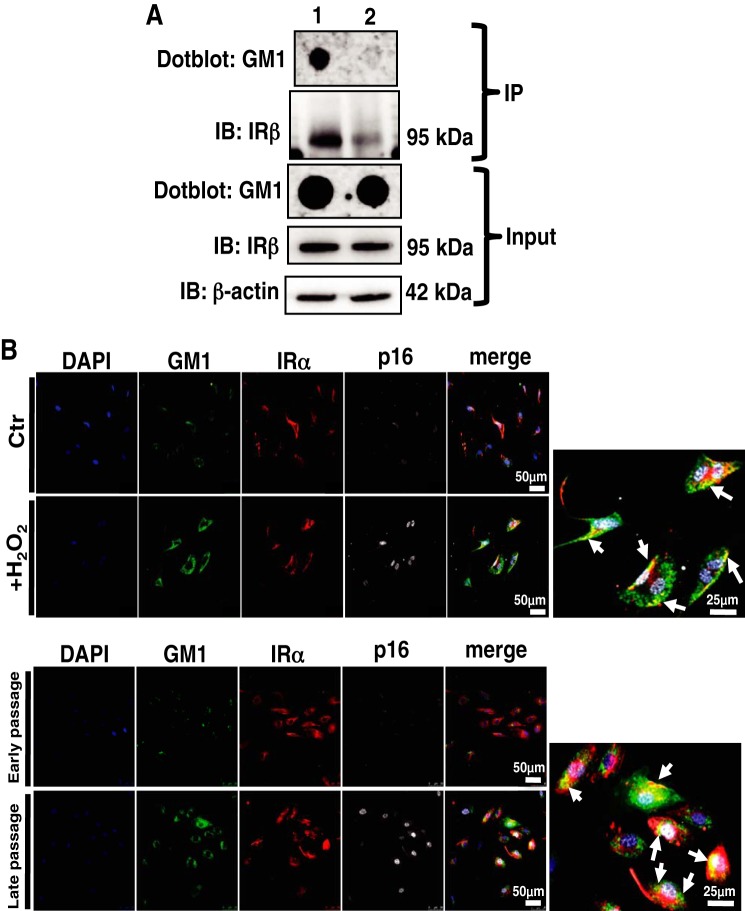
**Co-localization between GM1 and IR in senescent ECs.** *A*, whole-cell lysates from HAECs were immunoprecipitated (*IP*) with anti-IRβ antibody (*lane1*). Control immunoprecipitations were performed using heat-inactivated anti-IRβ antibody (*lane 2*). Immunoblotting (*IB*) was performed in the immunoprecipitates for GM1 and IRβ. Representative images are shown. *B*, immunocytochemical staining was performed in non-senescent and H_2_O_2_-induced prematurely senescent HAECs and early- and late-passage HAECs. Representative images are shown. (IRα, *red*; GM1, *green*; p16, *gray*; DAPI, *blue*). *White arrows* show co-localization of GM1 and IR. Control (*Ctr*): untreated cells (non-senescent).

It has been demonstrated that interaction between GM3 and IR is required for insulin resistance (26). Therefore, we examined whether GM1 and IR interacted in senescent HAECs. Using an immunoprecipitation approach, we found that GM1 and IR were physically associated in whole-cell lysates prepared from HAECs (Fig. 5*A*). No immunoprecipitation was seen when heat-inactivated antibodies were used (Fig. 5*A*). Thus, these results clearly indicate that GM1 has the ability to interact with IR in HAECs. Next, immunocytochemical staining showed that GM1 on senescent HAECs was closely co-localized with cell surface IR (Fig. 5*B*). On the basis of these results, it is suggested that the increased abundance of GM1 on senescent ECs contributes to insulin resistance through the interaction of GM1 with IR.

##### Increased Abundance of Ganglioside GM1 Contributes to Insulin Resistance

**FIGURE 6.**
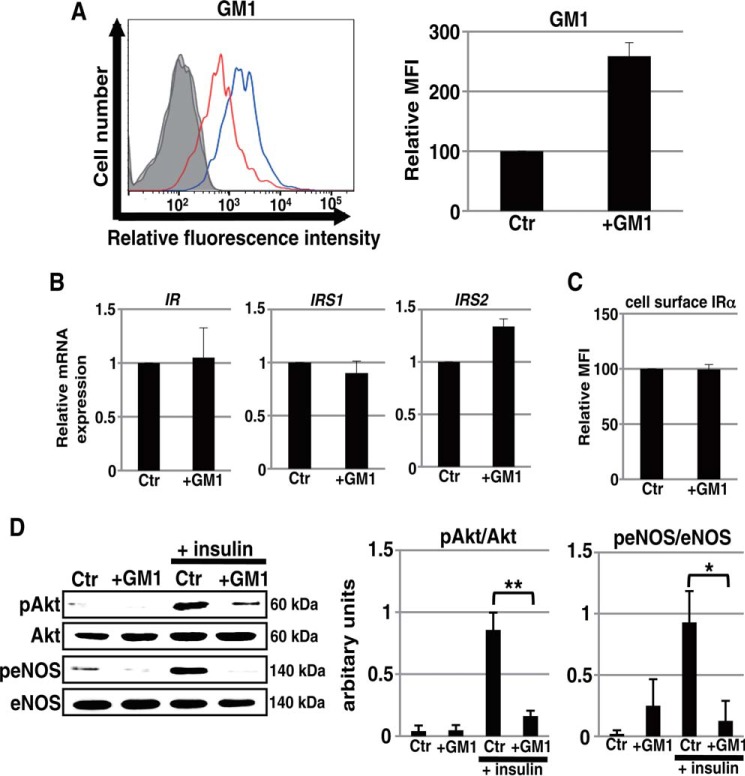
**Contribution of an increased abundance of GM1 to insulin resistance in ECs.** *A*, cell surface abundance of GM1 in non-senescent HAECs cultured with or without exogenous GM1 was analyzed by flow cytometry. Three independent experiments were performed, and representative overlaid histogram is shown at *left* (*red* and *blue lines* represent the results for non-senescent HAECs without and with GM1 treatment, respectively). Controls are shown by *thinner lines* with *gray* color. MFIs relative to that of non-senescent HAECs without GM1 treatment of three independent experiments are shown. *B*, real time PCR analysis of *IR*, *IRS1*, and *IRS2* was performed using cDNA derived from control and exogenous GM1-treated HAECs. The results shown were normalized against the values obtained for non-treated control HAECs (value = 1). Results are presented as means and S.D. of three independent experiments. *C*, FACS analysis for cell surface IRα was performed in control and exogenous GM1-treated HAECs. MFIs relative to that of non-treated control HAECs of three independent experiments are shown. *D*, Western blot analysis for insulin signaling was performed in non-senescent HAECs cultured with or without exogenous GM1. The *histograms* show mean densitometric readings ± S.D. for the phosphorylated proteins normalized to those of the loading controls. Values were obtained from three independent experiments; *, *p* < 0.05; **, *p* < 0.01. Control (*Ctr*): untreated cells.

To confirm that an increased abundance of GM1 contributes to insulin resistance, we investigated insulin signaling in HAECs incubated with exogenous GM1. As shown in Fig. 6*A*, 24 h of incubation with exogenous GM1 resulted in an increased abundance of GM1 in the cell membrane of early-passage HAECs. This was not accompanied by significant changes in the expression levels of IR and IRS (Fig. 6, *B* and *C*). Western blot analysis showed that insulin signaling was significantly reduced in HAECs incubated with exogenous GM1 (Fig. 6*D*). This result clearly demonstrates that an increased abundance of GM1 in the cell membrane specifically contributes to insulin resistance in ECs.

##### Abundance of Ganglioside GM1 Increases with Aging in the Cell Membrane of ECs

**FIGURE 7.**
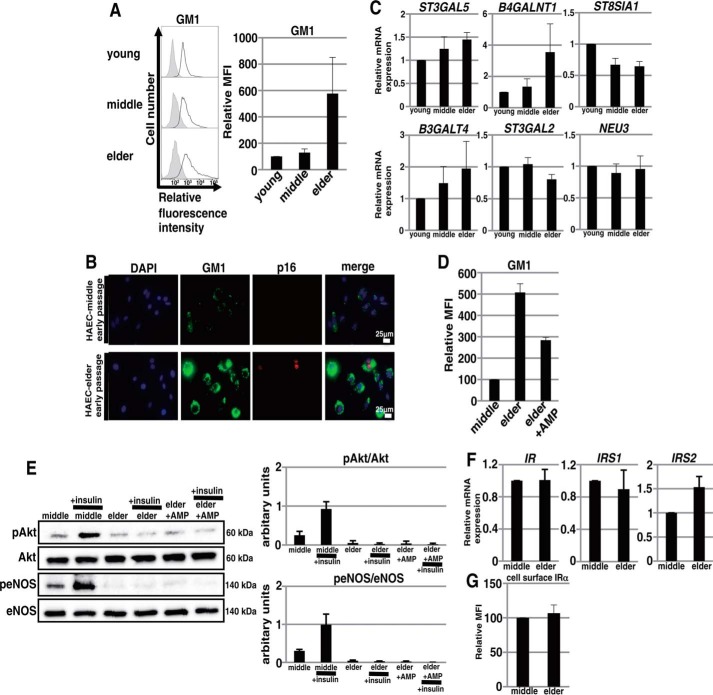
**Increased abundance of GM1 with aging and insulin resistance in ECs derived from older people.** *A*, cell surface abundance of GM1 in three HAEC cultures (HAECs-young, HAECs-middle, and HAECs-elder) was analyzed by flow cytometry at early passage (the same passage numbers as those of newly purchased cell stocks). Three independent experiments were performed, and representative results are shown. Controls are presented by *thinner lines* with *gray* color. MFIs relative to that of HAECs-young of three independent experiments are shown. *B*, immunocytochemical staining was performed in HAECs-middle and HAECs-elder at early passage. Representative images are shown (p16, *red*; GM1, *green*; DAPI, *blue*). *C*, real time PCR analysis of the glycosyltransferases shown in Fig. 1*B* and *NEU3* was performed using cDNA derived from three HAEC cultures derived from subjects of different ages, HAECs-young, HAECs-middle, and HAECs-elder, at early passage (the same passage numbers as those of newly purchased cell stocks). The results shown were normalized against the values obtained for HAECs-young (value = 1). Results are presented as means and S.D. of three independent experiments. *D*, cell surface abundance of GM1 in HAECs-middle and HAECs-elder with and without AMP-dNM treatment for 3 days was analyzed by flow cytometry at early passage. MFIs relative to that of HAECs-middle of three independent experiments are shown. *E*, Western blot analysis for insulin signaling was performed in HAECs-middle and HAECs-elder with and without AMP-dNM treatment for 3 days at early passage. The *histograms* show mean densitometric readings ± S.D. of the phosphorylated proteins normalized to those of the loading controls. Values were obtained from three independent experiments. *F*, real time PCR analysis of *IR*, *IRS1*, and *IRS2* was performed using cDNA derived from HAECs-middle and HAECs-elder at early passage. The results shown were normalized against the values obtained for HAECs-middle (value = 1). Results are presented as means and S.D. of three independent experiments. *G*, FACS analysis for cell surface IRα was performed in HAECs-middle and HAECs-elder at early passage. MFIs relative to that of HAECs-middle of three independent experiments are shown.

As demonstrated above, GM1 increases in abundance with cellular senescence and contributes to insulin resistance in senescent ECs. Next, we examined whether aging also contributes to increased GM1 abundance resulting in insulin resistance of ECs. We first compared the abundance of GM1 among HAECs derived from subjects of three different ages. FACS analysis showed that the abundance of GM1 in HAECs-elder, which were derived from an 81-year-old, was significantly higher than that of HAECs derived from younger subjects, even at early passage (Fig. 7*A*). Furthermore, immunocytochemical staining showed that GM1 was highly expressed in HAECs-elder (Fig. 7*B*). Among glycosyltransferases involved in the GM1 synthetic pathways, real time PCR analysis showed that *ST3GAL5*, *B4GALNT1,* and *B3GALT4* were expressed at higher levels in HAECs-elder than in HAECs derived from younger subjects (Fig. 7*C*). Thus, these results indicate that the abundance of GM1 is increased with aging in ECs. Next, we compared insulin signaling between HAECs-middle (40-year-old) and HAECs-elder (81-year-old) at early passage. As shown in Fig. 7*E*, insulin-mediated Akt and eNOS activation were not observed in HAECs-elder but were detected in HAECs-middle, despite the expression levels of IR and IRS showing no significant difference between these HAECs (Fig. 7, *F* and *G*). These results suggest that the high abundance of GM1 in HAECs-elder contributed to their insulinresistance. However, AMP-dNM treatment did not restore insulin signaling in HAECs-elder (Fig. 7*E*). AMP-dNM treatment lowered the abundance of GM1 on HAECs-elder (Fig. 7*D*), but it was not fully reduced to the same level as that of the non-senescent HAECs-middle. This is suggested to be one of the reasons why the restoration of insulin signaling in HAECs-elder was not observed after AMP-dNM treatment. Collectively, these data indicate that the abundance of GM1 is increased with aging in ECs and suggest that an increased abundance of GM1 occurs with aging and contributes to insulin resistance in the ECs of older people.

## Discussion

Insulin signaling in ECs plays important roles in the maintenance of vascular integrity. Therefore, the development of insulin resistance in ECs leads to increased vascular morbidity and mortality. The remaining issues are the molecular mechanisms of the insulin resistance that occurs with senescence and aging. In this study, we have demonstrated that the abundance of GM1 increases on the cell surface of senescent and aged ECs and contributes to insulin resistance in senescent ECs. Therefore, we propose that the increased abundance of GM1 in ECs with senescence and aging is a risk factor for vascular diseases in older people.

GM1 is synthesized by glycosyltransferases and sialidase (*NEU3*) as shown in Fig. 1*B*. The changed expression of these enzymes leads to an increased production of GM1. In fact, the overexpression of *B4GALNT1* and *B3GALT4* or *NEU3* in mammalian cells was reported to induce an increase in the abundance of GM1 (24, 27). In senescent ECs, we found that the expression of *B4GALNT1*, which catalyzes the synthesis of GM2 (a GM1 precursor), was up-regulated. Furthermore, a decrease in the abundance of GD3 was observed in senescent ECs, suggesting that the GM2 and GM1 synthetic pathway is predominant in senescence. These results are suggested to be one explanation for the increased abundance of GM1 in senescent cells. In aged ECs (HAECs-elder), an increased abundance of GM1 (relative mean fluorescent intensity (MFI) ∼500) was detected, compared with that in senescent HAECs-middle (relative MFI ∼200). Also, three glycosyltransferases involved in GM1 synthetic pathways, *ST3GAL5*, *B4GALNT1,* and *B3GALT4*, were up-regulated in the aged ECs. Aging may be accompanied by altered expression levels of these enzymes. Further study should be required to clarify the mechanisms regulating the expression of these enzymes and to identify the key regulators of GM1 abundance in ECs with senescence and aging.

As shown in Fig. 3, *D* and *H*, the expression levels of IR on the cell surface were slightly reduced in senescent ECs. This reduction in IR expression was restored after AMP-dNM treatment, which lowered the increased GM1 levels in senescent ECs (data not shown). Furthermore, exogenous GM1 treatment for 3 days induced a slight reduction in the expression of IR on the cell surface of ECs, which was similar to that observed in senescent ECs (data not shown). It has been reported that the cell surface abundances of gangliosides affect the expression levels of raft-associated proteins (28). Thus, it is suggested that increased GM1 may affect the expression of IR on the cell surface, although the molecular mechanism underlying the regulation of cell surface IR via GM1 is unknown. In contrast, 24 h after treatment of ECs with exogenous GM1, insulin resistance was induced without any reduction in the expression of IR on the cell surface (Fig. 6*C*). Therefore, this result indicates that a slight reduction in the expression of IR on the cell surface does not affect insulin signaling in senescent ECs and confirms that an increased abundance of GM1 functionally contributes to insulin resistance with senescence.

**FIGURE 8.**
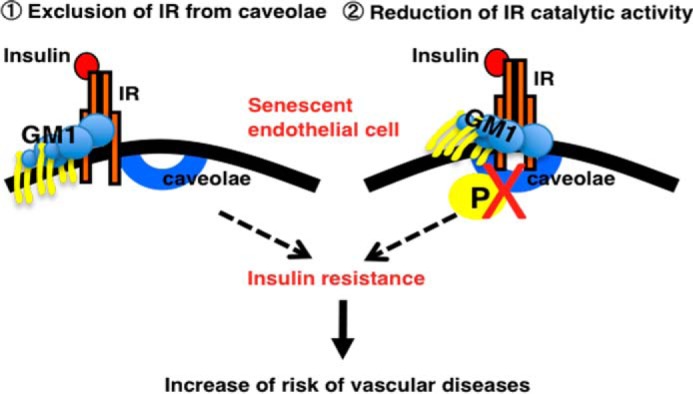
**Two proposed mechanisms to explain how an increased abundance of GM1 can increase insulin resistance in senescent ECs**. *1*) exclusion of IR from caveolae; GM1 interacts with IR followed by the dissociation of IR from caveolae, resulting in insulin resistance; *2*) reduction of IR catalytic activity; increased GM1 interacts with IR and then directly affects IR kinase activity, resulting in insulin resistance.

In this study, we have demonstrated that increased GM1 contributes to insulin resistance in senescent ECs, presumably due to a physical association between GM1 and IR. It has not been established how increased GM1 regulates insulin signaling in ECs, but we propose two possible mechanisms (Fig. 8). One is that an association between the GM1 carbohydrate moiety and an epitope in the extracellular domain of IR leads to the exclusion of IR from caveolae, as was recently found for ganglioside GM3 and IR (26). The second proposed mechanism is that an increased abundance of GM1 affects the catalytic activity of IR and attenuates signal transduction, as has been suggested by previous reports (24). To clarify the molecular mechanism underlying the development of insulin resistance via an increased abundance of GM1 in ECs, further study is required.

In adipocytes, TNFα treatment was reported to induce an increased abundance of GM3 in the cell membrane, and this increased abundance of GM3 was found to produce insulin resistance (15). In this study, we have demonstrated that an increased abundance of GM1 produces insulin resistance in ECs. We also investigated the abundances of gangliosides in ECs after TNFα treatment. In ECs, GM1 was increased but GM3 was not.^3^ Furthermore, in transgenic mice showing insulin resistance, *NEU3* overexpression induced increases in the abundances of GM1 and GM2 in several tissues, including liver (24). Thus, it is possible that the abundances of gangliosides related to insulin resistance differ among cell types and tissues. So, clarifying the significance of the abundance of each ganglioside in relation to tissue-specific insulin resistance could lead to a deeper understanding of each pathological condition and thus to more efficient drug discovery for the treatment of insulin resistance-related diseases.

Beneficial effects of AMP-dNM on pathological model mice have been reported. AMP-dNM treatment restores insulin sensitivity in *ob*/*ob* mice (16) and also inhibits atherosclerosis in APOE*3 Leiden as well as low-density lipoprotein receptor^−/−^ mice (29). In the former report (16), it was suggested that reducing the increased abundance of GM3 in adipocytes by AMP-dNM treatment improves insulin sensitivity. In the latter report (29), lowering of plasma cholesterol by AMP-dNM treatment was proposed to reduce the development of atherosclerosis. Recently, it has been demonstrated that insulin resistance in ECs plays major roles in type 2 diabetes and cardiovascular diseases (4, 5). In this study, we have demonstrated that increased GM1 contributes to insulin resistance in ECs. It is considered that an increased abundance of GM1 on ECs occurs in pathological conditions such as obesity and atherosclerosis, and it has been reported that senescent ECs are present in atherosclerotic lesions (30). Therefore, we speculate that the reduction of increased GM1 abundance by AMP-dNM treatment could also contribute to the improvement of insulin resistance-related pathological conditions.

Increased GM1 is known to affect the cell surface expression of raft-associated proteins and to contribute to the reduction of membrane fluidity (27, 31, 32). Several cellular dysfunctions, such as impaired signal transduction, etc., are considered to be due to changes in the composition of gangliosides in the cell membrane. In this study, we have shown that the abundance of GM1 is increased on senescent ECs, resulting in insulin resistance. Furthermore, it is possible that an increased abundance of GM1 on ECs with senescence may also cause other EC dysfunctions, although further studies are required to investigate this.

Taken together, as described above, we have shown that the abundance of GM1 on the surface of ECs is increased with senescence and aging. We suggest that an increased abundance of GM1 in cell membranes may produce insulin resistance throughout the body, resulting in the initiation and/or progression of serious vascular diseases. Notably, an increased abundance of GM1 with senescence and aging may contribute to the elevated risk of vascular diseases in older people. We propose that GM1 could be an attractive target for the detection, prevention, and treatment of insulin resistance, particularly in older people. The inhibition of GM1 synthesis and the interaction between GM1 and IR may offer avenues for the future development of new therapeutic strategies. To facilitate the clinical translation of these findings, further study will be required to identify the key regulators of GM1 homeostasis and clarify the molecular mechanism underlying the production of insulin resistance via an increased abundance of GM1.

## Author Contributions

N. S. designed and performed experiments, analyzed data, and wrote all of the paper. Y. I. analyzed data. M. T. designed experiments and wrote the paper with N. S. All authors reviewed the results and approved the final version of the manuscript.

## Footnotes

3 N. Sasaki, Y. Itakura, and N. Toyoda, unpublished data.

## Footnotes

2 The abbreviations used are:

EC: endothelial cell
eNOS: endothelial NO synthase
GSLs: glycosphingolipids
GM3: monosialodihexosylganglioside
GM1: monosialotetrahexosylganglioside
HAEC: human aortic endothelial cell
EGM-2: endothelial growth medium-2
PDL: population doubling level
AMP-dNM: *N*-(5′-adamantane-1′-yl-methoxy)-pentyl-1-deoxynojirimycin
SA-β-Gal: senescence-associated β-galactosidase
GM2: monosialotrihexosylceramide
GD1a: disialogangliotetraosylceramide
IR: insulin receptor
IRS: IR substrate
MFI: mean fluorescent intensity.
